# Self-Management Maintenance Inhalation Therapy With eHealth (SELFIE): Observational Study on the Use of an Electronic Monitoring Device in Respiratory Patient Care and Research

**DOI:** 10.2196/13551

**Published:** 2019-05-30

**Authors:** Esther Kuipers, Charlotte C Poot, Michel Wensing, Niels H Chavannes, Peter AGM de Smet, Martina Teichert

**Affiliations:** 1 Department of IQ Healthcare Radboud Institute for Health Sciences Radboud University Medical Centre Nijmegen Netherlands; 2 BENU Apotheek Zeist West Zeist Netherlands; 3 Department of Public Health and Primary Care Leiden University Medical Centre Leiden Netherlands; 4 Department of General Practice and Health Services Research University Hospital Heidelberg Heidelberg Germany; 5 Department of Clinical Pharmacy Radboud Institute for Health Sciences Radboud University Medical Centre Nijmegen Netherlands; 6 Department of Clinical Pharmacy & Toxicology Leiden University Medical Centre Leiden Netherlands

**Keywords:** eHealth, pharmacy, inhalation therapy, asthma, COPD, pharmacy practice research

## Abstract

**Background:**

Electronic inhalation monitoring devices (EIMDs) are available to remind patients with respiratory diseases to take their medication and register inhalations for feedback to patients and health care providers as well as for data collection in research settings.

**Objective:**

This study aimed to assess the validity as well as the patient-reported usability and acceptability of an EIMD.

**Methods:**

This observational study planned to include 21 community pharmacies in the Netherlands. Patient-reported inhalations were collected and compared to EIMD registrations to evaluate the positive predictive value of these registrations as actual patient inhalations. Patients received questionnaires on their experiences and acceptance.

**Results:**

A convenience sample of 32 patients was included from across 18 pharmacies, and 932 medication doses were validated. Of these, 796 registrations matched with patient-reported use (true-positive, 85.4%), and 33 inhalation registrations did not match with patient-reported use (false-positive, 3.5%). The positive predictive value was 96.0%, and 103 patient-reported inhalations were not recorded in the database (false-negative, 11.1%). Overall, patients considered the EIMD to be acceptable and easy to use, but many hesitated to continue its use. Reminders and motivational messages were not appreciated by all users, and more user-tailored features in the app were desired.

**Conclusions:**

Patients’ interaction with the device in real-world settings is critical for objective measurement of medication adherence. The positive predictive value of this EIMD was found to be acceptable. However, patients reported false-negative registrations and a desire to include more user-tailored features to increase the usability and acceptability of the EIMD.

## Introduction

Electronic monitoring devices are increasingly important in the self-management of chronic diseases such as chronic obstructive pulmonary disease and asthma. These two chronic respiratory diseases affect an estimated 384 and 235 million people worldwide, respectively [1,2]. According to the prevailing clinical guidelines, daily intake of inhaled corticosteroids is the cornerstone for optimal asthma treatment, and inhaled corticosteroids are also used in chronic obstructive pulmonary disease, together with bronchodilators [2,3].

An electronic inhalation monitoring device (EIMD), which measures inhalation actuations, provides detailed data on patient adherence to treatment for both patient and health care providers [4-7]. EIMDs, in combination with audiovisual reminders and feedback, have the potential to improve medication adherence and health care outcomes by facilitating self-management and aiding in clinical (shared) decision making [8-15]. Previous studies have shown that involving patients in monitoring their own symptoms can lead to improved awareness and competence in disease management [16].

Besides clinical practice, EIMD data could be used as objective outcome measures for medication adherence in research [17-19]. The integrated time stamp enables comprehensive data collection in research populations on the timing and pattern of inhaler actuation, including dose dumping [20]. This way of data collection was stated to be more accurate and objective for the evaluation of medication use and is considered to be superior to self-report, canister weighing, or pharmacy dispensing data [17,21].

However, the widespread use of EIMDs for measuring patients’ medication adherence in clinical practice and research setting depends on the acceptability by patients as well as health care providers and researchers, respectively. From the perspectives of both patient care and research, it is important that the EIMD is accurate and valid [20,22-24]. Earlier in-vitro studies (product validation studies) evaluating the validity of an EIMD, following a fixed protocol and simulating inhalations, found 99.2% overall accuracy of detection [25]. On the contrary, small-scale studies reported malfunctioning devices and potential loss of data as well as overrecording of doses that did not reflect actual inhalations [22-24,26]. Use of EIMDs as objective measures for medication adherence for research purposes in a real-world setting depends not only on technical capabilities, but also on how the user interacts with the system in real life. In other words, results in laboratory settings might not apply to EIMD validity and reliability in the broad use by community-dwelling patients. As EIMDs could be used for measuring real-time medication adherence in clinical trials, evidence on their potential to measure patient inhalations in community-dwelling patients is relevant.

For the implementation of EIMD in clinical practice, it is important to acknowledge the complexity of the implementation process, influenced by patients’ acceptance and ability to use information technology tools [27,28]. Hence, before the EIMD is implemented and used as an accurate and reliable measure for patients’ actual medication adherence in a real-world setting (in both clinical decision making and research), a rigorous evaluation of the technical performance, usability, and acceptability in clinical practice is required.

This study aimed to assess the validity and patient-reported usability and acceptability of an electronic adherence monitor and reminder device for patient care and research.

## Methods

### Study Design

This prospective observational study on agreement between EIMD measurements and patient-reported inhalations was conducted between April 18 and May 25, 2018.

### Setting

This study was conducted by 21 students in the second year of their master’s program in pharmacy from the University of Leiden, the Netherlands, during their internships in community pharmacies. The students were asked to validate EIMD registrations for two patients each, for 3 weeks, and they received additional training on the use of the EIMD program. The individual monitoring of patients was time consuming because of the protocol-specific information requirements, and consequently, dedicated persons with enough time were needed in the pharmacy. Additionally, the acquaintance with an innovative device to be implemented into daily practice and individual patient coaching were relevant learning objectives for the students.

Dutch pharmacists have a professional and legal responsibility to provide drug treatment for their patients and, as most patients in the Netherlands attend one community pharmacy, pharmacists usually possess the complete medication histories of their patients [29].

### Ethics Approval

The study protocol was approved by the Ethical Committee of the Radboudumc Nijmegen (approval number, 2018-4153). Written informed consent was obtained from all individual participants included in this study, prior to the study.

### Electronic Monitoring

The EIMD to be assessed was the Turbu+ V2.1 (AstraZeneca UK Limited), consisting of three components: (1) the electronic device that could be attached to the inhaler of the corresponding product “Symbicort Turbuhaler,” (2) an app to be installed on the patient’s mobile phone, and (3) an online portal allowing the health care professional access to the same actuation data. During patient inhalation, the device was actuated and the date and time of the actuations were recorded. The data were synchronized with the app on the mobile phone by Bluetooth, and the app visualized the timeline of these data up to the previous month. All EIMD data for measured actuations were automatically and electronically linked to the online portal of the health care provider for his/her patients and an additional research database containing the same data but anonymized. The research database has been setup to study patterns of medication adherence and to evaluate the effectiveness on interventions for medication adherence across multiple studies. Actuation data from all health care professionals included in this study were aggregated into one central research database containing data on all included patients from the participating pharmacies.

Patients could only use the EIMD program after they were enrolled and trained by their health care provider. After entering the name, birth of date, sex, dose regimen settings, and email address, the patient automatically received an email with the link to download the app. The time(s) for a pop-up reminder in the app could be set individually by the patient at the time of scheduled inhalation(s). If patients did not take their medication, 30 minutes after the scheduled inhalation, a “missed-medication” motivational message was sent automatically. Patients received a weekly motivational push notification in the app (eg, “Great week. You’ve been following your prescription this week! Keep it up!”).

### Patient Inclusion

Patients were eligible to participate if they (1) were current users of budesonide/formoterol Turbuhaler (Anatomic Therapeutic Chemical code R03AK07) [30] with at least two prescriptions in the previous 12 months; (2) were of age ≥ 18 years; (3) were regular patients in the pharmacy (registered in the pharmacy system and receiving dispensings from this pharmacy only); (4) had access to a smartphone, and (5) were able to use the internet. We aimed to include current users who were familiar with the inhaler. At random, eligible patients were invited during regular pharmacy visits or by phone. If interested, patients received an information leaflet on the study and an informed consent form. Patients interested in participating were asked to provide informed consent, allowing the student to collect general data about gender, age, use of short corticosteroid courses (indicating exacerbations), and refills from the pharmacy database (to assess adherence rates). Eligible patients were invited to the pharmacy for an intake visit.

### Intake Visit

During the intake visit, the students provided the patient with an EIMD and (oral and written) instructions. The patients attached the device to the inhaler and installed the app during the visit or at home following the instructions. Additionally, the students verified the pharmacy’s information collected on drug use, patient’s refill adherence to inhaled corticosteroids, and the number of oral corticosteroid courses in the previous year. Subsequently, the patients completed the Beliefs About Medicines Questionnaire (BMQ), consisting of two parts: the BMQ Specific list, which measures perceptions of specific medicines, and the BMQ General list, which measures general beliefs about medicines. The BMQ Specific list comprises two scales—one assessing patients’ beliefs about the necessity of preventer medication for maintaining present and future health (Necessity scale) and the other for assessing their concerns about the potential adverse consequences of using such medication (Concerns scale) [31,32]. All items were rated on a 5-point Likert scale, with a range of 5-25 possible scores for both scales.

The students explained that they would call the patients 6 times within 14-21 days to check on their actual drug intake on the previous days and agreed on the most suitable days and time to call. If patients wished to, they were allowed to use a paper diary.

### Accuracy and Patient-Reported Use of the Electronic Inhalation Monitoring Device: Procedures and Measures

During the follow-up period of 14-21 days, the students phoned the patients 6 times to check on their actual drug intake and EIMD performance from the previous day, and if possible, up to the day of the earlier phone call. The student collected and reported information on the number and time of daily inhalations, and all particularities or other circumstances that the patient reported were registered by the student in an Excel file. These were subsequently centralized into one patient self-reported database.

To avoid socially desirable answers and to collect reliable data, patients were instructed that this study focused on the accuracy of the device (instead of their medication use), and they were asked to use the medication at their usual dose and frequency and to use only the EIMD prepared device during the study period and no other inhalers of this medication. Semistructured questions were used during the phone call; for example, “When did you use your medication for the last time?” and “Do you see any registrations in your app that do not represent real medication use?”

During the phone call, the student had immediate access to the EIMD data and compared the data from the research database with the information reported by the patient (and registered in the patient self-reported database). Any discrepancies were directly discussed with the patient and registered.

### Usability and Acceptability of the Patient Electronic Inhalation Monitoring Device: Procedures and Measures

To evaluate patients’ usability, patients received two questionnaires at the end of the study, including the System Usability Scale (SUS), which is a validated instrument for evaluating the usability of a wide range of products and services [33,34]. The SUS score can range from 0 to 100. For products with a score less than 70, improvement options should be considered [34].

The second questionnaire addressed the experiences and acceptability of the program and the expectations on the pharmacists’ role in counselling. This part consisted of 12 statements, formulated positively or negatively, measured on a 5-point Likert Scale (1=strongly disagree, 5=strongly agree), followed by 3 multiple-choice questions regarding the frequency of using the app and whether the patient would like to continue the program and by 4 open questions on advantages, disadvantages, and targets for improvements.

### Data Analysis

The main outcome measure was the positive predictive value (PPV), calculated as (the number of correctly registered doses according to the patient self-reported database and registered database)/(the total number of registered doses [true and false positives])*100. Results of the questionnaires were analyzed using descriptive statistics. Responses to the open questions were coded and summarized for patients’ opinions, wishes, and barriers encountered using the EIMD program to identify key topics via the grounded theory approach [35].

Patient-specific characteristics were assessed with the scores for the BMQ Specific and BMQ General questionnaires, represented on a continuous scale. The BMQ necessity and concerns scores were split at the scale midpoints to distinguish between four subgroups: accepting (high necessity, low concerns), ambivalent (high necessity, high concerns), skeptical (low necessity, high concerns), and indifferent (low necessity, low concerns) [36]. Refill adherence was calculated as the proportion of days covered in the prior 12 months (due to prior dispensings up to 15 months).

## Results

### Patient Demographics and Questionnaire Completion

A total of 32 patients were included by 18 students (Table 1). In 3 pharmacies, no patients eligible and willing to participate were available. The mean age of the included patients was 48.1 years (range, 20-69 years), and 17 patients were female (53.1%). Medication adherence in the previous year calculated from dispensing data was 81.3%. Most patients used their medication twice a day (n=26). Three patients (9%) used an additional diary to note their daily inhalations. Twenty-five patients (78%) completed the questionnaires and provided individual comments on the EIMD (Table 2 and Textbox 1).

**Table 1:** Patient characteristics (N=32).

Characteristic	Value
Age (years), mean (range)	48.1 (20-69)
Female, n (%)	17 (53.1)
Number of oral corticosteroid courses in the last year, mean (range)	0.31 (0-4)
Medication adherence in the previous year calculated from dispensing data (n=28), mean % (range)	81.32 (17.53-194.80)
BMQ^a^ General Harm score (n=29; possible range: 4-20), mean (range)	10.21 (5-16)
BMQ General Overuse score (n=29; possible range: 4-20), mean (range)	8.04 (4-13)
BMQ Specific Necessity score (n=30; possible range: 5-25), mean (range)	17.70 (10-24)
BMQ Specific Concerns score (n=30; possible range: 5-25), mean (range)	10.97 (6-17)

^a^BMQ: Beliefs About Medicines Questionnaire.

**Table 2:** Patients’ acceptability of the Electronic Inhalation Monitoring Device (EIMD) program. Values in italics indicate the highest percentage(s) per statement.

Statement	Disagree (score 1-2), n (%)	Neutral (score 3), n (%)	Agree (score 4-5), n (%)
The EIMD programme does provide useful insights in my medication use^a^	*11 (44)*	3 (12)	*11 (44)*
The EIMD programme is useful to me	6 (24)	8 (32)	*11 (44)*
The EIMD programme is time-consuming	*19 (76)*	2 (8)	4 (16)
The motivational messages in the EIMD app feel positive for me	*11 (44)*	10 (40)	4 (16)
The reminders of the EIMD programme help me to take my medication in time	*10 (40)*	6 (24)	9 (36)
The EIMD programme contributes to dealing with my illness^a^	*10 (40)*	5 (20)	*10 (40)*
The EIMD programme contributes to the interaction with my pharmacist	*10 (40)*	8 (32)	7 (28)
The EIMD programme helps establishing a habit to use my medication	*11 (44)*	9 (36)	5 (20)
The EIMD programme gives me confidence to help manage my disease	*10 (40)*	9 (36)	6 (24)
The pharmacist’s monitoring of my medication use with this programme helps me to use my medication better^a^	*13 (52)*	6 (24)	6 (24)
When someone can monitor my medication intake, I take my medication as usual^a^	4 (16)	3 (12)	*18 (72)*
I see added value of the EIMD programme to manage my medication intake^a^	*11 (44)*	5 (20)	9 (36)
I would recommend the EIMD programme to other patients	7 (28)	*12 (48)*	6 (24)
The pharmacist can play an important role in counselling	2 (8)	3 (12)	*20 (80)*

^a^Item was phrased as a negative statement.

**Selection of patients’ free-text comments about the Electronic Inhalation Monitoring Device program. Values in brackets represent gender and age of the participants (eg, F24=female, 24 years).**

**Technical functionalities:**

If the app synchronises the data, this may take a long time (maybe this can be improved). In the end, the registration of my puffs did not go well.

F24

Until now, the app missed three inhalation registrations in the morning. I clicked twice within a minute, but only one [inhalation] was registered.

M52

**Patient-technology interaction:**

Not all inhalations were registered [in the app on my mobile phone], so I inhaled again and used too much medication.

F45

The app is not always working. If medication is taken, this is not always measured. Even if the medication has already been taken (before the time set), still a reminder was sent. The app does not provide insight into whether the medication has been taken in the right way.

F20

The alarm you can set will never go off. So you have no reminder.

F45

The Turbu+ did not register when the app was [physically] not around, and it was therefore not possible to register 2 inhalations in 1 day.

M55

The notifications did not work with my Galaxy s7 Edge.

M57

**Patients’ opinions and acceptability of the program:**

Regarding the device/app:

The device itself feels rather rickety. The two parts did not really fit together.

M51

The idea is good, I also see the commercial need, but think about the return for the patient. Information in the app for how many days medication is still available in the inhaler, would be such convenient return for the patient. For example, before he goes on holiday, he knows if there are extra medicines needed.

M55

I feel that tracking medication use can be useful for many patients. However, it remains very difficult, because the connection of the device and the app is not clear. As a result, it [medication use] is registered at some time, but not on other times, for unclear reasons. With this, possible useful options of the app will immediately disappear.

M43

Regarding reminders:

If you are not adherent, the Turbu+ can be convenient as a reminder for using the medication.

F39

[Useful] when you forget [medication intake] (but I never forget this).

M42

I personally liked the reminders, and it was also very pleasant that I could monitor myself whether I had taken it or not!

F24

I would like to modify the app myself for what I agreed on with my doctor (about minimum and maximum use per day).

F47

[I would prefer] a clearer reminder sound signal.

V56

Regarding motivational messages:

The tone of the written messages deserves carefulness: the patient is the boss, the app only gives advice.

F47

Regarding attitude to electronic monitoring:

I would only use the app if only I can see it [the data]. Watching by pharmacist should not be the default for each patient.

F47

I am intrinsically motivated to use my medication and do not really need an app for that.

M55

Once the routine is there to take an inhalation twice a day, it has little added value for the users. It gives the impression that it is only useful for the pharmacist and the manufacturer to collect Big Data.

M55

I always take my medication when brushing my teeth. So there was no need for help with the intake. In addition, I am able to feel when I have to use my medication more often, according to my needs. I think that the app can help people who have difficulties with this.

M28

[An advantage is] to check when I am not sure about forgotten medicines. Insights into patterns in periods when I have more symptoms help me to anticipate.

F48

Thirty patients (94%) completed the BMQ General and Specific questionnaires at the start of the study. The majority of them could be classified as acceptant (n=19) regarding their inhalation medication: 4 patients were ambivalent and 7 were indifferent. No patients were classified as skeptical.

### Accuracy and Patient-Reported Use of the Electronic Inhalation Monitoring Device

Of the 32 patients, 28 completed all 6 phone calls. Overall, the 18 students verified 932 medication doses (mean=29.1 doses verified per patient; range=3-88). A total of 796 doses registered in the research database matched patient-reported inhalations captured in the patient self-reported database (true-positive, 85.41%). In addition, 33 inhalations were registered in the research database that did not match the actual drug intake, as reported by the patients in the patient self-reported database (false-positive, 3.5%). Further, 22 patients reported an average of 4.7 inhalations (range, 1-20) that were not recorded in the research database, accounting for a total of 103 inhalations (false-negative, 11.1%).

Information on registration of 6 of the 32 devices was in full agreement with the patient-reported inhalations. The PPV of all registrations in the research database was 96.0%.Some patients reported technical problems with EIMD data recording and synchronization of the EIMD data with their mobile phone. Delayed data synchronization resulted in two patients taking more medication than prescribed under the assumption that they had forgotten their dose.

### Usability and Acceptability of the Electronic Inhalation Monitoring Device

The majority of patients indicated that the app was easy to use and not unnecessarily complex; the mean SUS score was 68.9 (SD 11.34; range, 52.5-90).

In the acceptability questionnaire, patients rated the EIMD generally as useful (76% neutral or agree) and 84% rated the EIMD program as not time-consuming (Table 2). With regard to recommending the EIMD to other patients, the majority rated this item as neutral (n=12, 48%). In addition, 80% of the patients indicated that they were not willing (n=15) or uncertain (“maybe,” n=5) about continuing the program themselves, and 70% (n=14) of these patients reported that they had only participated in this study because they wanted to help the students fulfil their assignment and facilitate the research. The five patients who were positive about continuation reported that they participated mainly to gain personal insight into their inhalation patterns. The patients generally felt positive about the pharmacists’ role in counselling.

Written feedback on the advantages, disadvantages, and targets for improvements was provided by 25 patients. This feedback was clustered into four themes: EIMD functionality, reminders, motivational messages, and attitude toward electronic monitoring (Textbox 1).

Some of the previously mentioned technical issues reported by some patients resulted in a level of frustration or confusion because of missed or unnecessary reminders due to missing data, which impacted their acceptance of the app (Textbox 1). The reminders and motivational messages were appreciated by 9 and 4 patients, respectively. Several patients suggested more individualization of the settings in the app, such as inclusion of a personal choice to share data with a specific health care provider and management of their dose regimen settings.

## Discussion

### Principal Findings

In this study, we found an acceptable PPV: 96.0% of the registered doses represented patient-reported drug intake. However, we found a high number of false-negative registrations: 11.1% of patient-reported inhalations were not recorded by the EIMD.

These unrecorded patient-reported inhalations could have been the result of a number of factors, either technical or user-related issues, that should be discussed. Although in earlier studies, loss of data or missing data were associated with technical issues such as battery drain, this was not likely to have occurred during the short duration of our study [7,24,26]. The high number of false-negative registrations could possibly be the result of overreporting by the patients or suboptimal use of the device (eg, Bluetooth not activated, EIMD not paired to the phone, or EIMD not within a 5-meter distance from the phone during inhaler actuation). Assessment of user experience revealed that the written instruction did not contain detailed information about the data synchronization protocol, which, combined with instructions at enrolment, may have led to some of the reported observations. In earlier research with the same EIMD, the researchers had presumed the possibility of false-negative as well as false-positive registrations, but they were unable to verify the registrations by patients’ actual inhalations or the data on user interaction with the system to interpret their findings [15].

This study demonstrated the importance of validating medication adherence data in real-world settings. Patients’ interaction with the device is critical for objective measurement of medication adherence in research and clinical settings. We emphasized on the importance of evaluating technical performance to identify technical/user issues and stressed the need of evaluating usability and acceptability across multiple components of the EIMD.

Previous studies on EIMD performance focused on accuracy and reliability in laboratory settings and lacked data collected in a real-life setting where patients interact with the EIMD. Furthermore, previous studies did not access patient acceptability and user experience [7,20,23,24,26,37,38], both of which are essential for successful implementation and sustained use in daily practice [39]. This is the first study to demonstrate the importance of evaluating and validating EIMDs in a real-world setting. The accuracy of inhalation measurement is essential from not only a research point of view, but also a clinical perspective, as false-negative registrations lead to unnecessary signals and reminder messages. Furthermore, it falsely reports patients as nonadherent, and this could lead to underestimation of adherence, incorrect clinical decision making, and overuse of medication when patients assume to have forgotten their dose.

### Patient Experiences and Acceptability of the Electronic Inhalation Monitoring Device

Our findings on acceptability and user experiences further underline the importance of evaluating EIMDs on acceptability, preferably early in the implementation process. There is a growing body of evidence on electronic health apps, in general, that do not perform as expected in clinical practice, because the app turns out to be unacceptable or does not fit the users’ needs [40-43]. Although patients indicated that the app was easy to use and not complex, the majority did not intend to use the app in the future, apparently because there was no clear personal need to use the EIMD. They were primarily motivated to use the EIMD to facilitate the research rather than having an intrinsic motivation to gain personal insights into their inhalation patterns, and their medication adherence in the previous year calculated from dispensing data was already high. Patients with intrinsic motivation to improve their disease management (eg, based on low adherence or impaired disease control) would possibly benefit more from the EIMD. Therefore, understanding different types of patient segments is important to succeed in the implementation; the EIMD needs to match with the patient profile (eg, adherence and asthma control), needs, and preferences. In addition, the fact that the majority of patients did not intend to continue with the EIMD may have been the result of a suboptimal technical performance, for example, loss or troubles with the Bluetooth connection, which was regularly experienced; delay in the synchronization of data from the EIMD to the mobile app; and inhalations taken just before midnight not visible on the intended day. These technicalities can probably easily be improved and thereby increase the chance of acceptance and successful implementation. Moreover, health care providers and patients would benefit from further development of the EIMD, so that it can not only detect inhaler actuation, but also check the inhalation quality, breath force, and inhalation technique.

Our findings emphasized the need for clear patient selection and a more individualized, tailored device. In this study, half of the people found the reminders helpful in taking the medication on time, while the other half found them useless. We found similar results for the motivational messages. Hence, when designing a self-management intervention containing multiple self-management strategies (ie, motivational messaging, reminders, and audio-visual behavioral feedback), it is important to critically review each component on usability separately and preferably tailor the intervention to the needs of the patient.

### Strengths and Limitations

This study has some limitations. First, the actual inhalations were self-reported by the patients, which could have led to bias by overreporting actual use (and thus also an overestimation of unrecorded actuations), as patients might tend to provide socially desirable answers. However, as a 24-hour patient observation was not feasible, this was the best way to collect data on patients’ actual drug intake within this setting. To prevent socially desirable answers, patients were instructed at the intake that this study was on verifying the registrations of the EIMD rather than their medication use or adherence, and during the phone calls, the students asked open questions. Additionally, patients were questioned about their actual inhalation mainly at the same and previous days, with a maximum of 5 days prior. Thus, the phone calls for data evaluation focused on the most recent moments of drug intake in order to reduce possible recall bias. In further analysis regarding the possible impact of recall bias, we did not observe any differences between measurements on day 5 and those on the earlier days, or between different age groups (data not shown). As earlier studies have shown that there is considerable variation in the accuracy of diaries to note medication intake [44], the use of a diary was not mandatory. As patients frequently reported technical issues as a possible explanation for both missing and extra registered inhalations, we do not expect much bias from this setting.

Second, all pharmacies were related to the master’s education program of pharmacy from the University of Leiden. Data were collected in different pharmacies and by different students, to prevent bias from specific settings. However, such bias could not be fully excluded. Although the students were not yet registered health care providers, they were quite motivated and technically skillful. The students selected a convenience sample of patients, although it seemed difficult to find patients with the original inhalation medication that was fit for the EIMD; in some pharmacies, no patients could be selected at all. Third, the short inclusion time of this study, due to the internship period, could have led to selection of patients who were more willing to help the students with their task rather than being interested in their own medication performance or adherence. This was reflected in patients’ individual comments: They regularly indicated that the program could be especially useful for other patients, but that it was of little value to them. Some patients reported that they did not need “help with the inhalation” or “an app for taking the medication.” With a mean medication adherence of >80%, this seems to be a group with relatively high adherence and could indicate selection bias of the more adherent patients, for which the device may be less useful. Further research is needed on how health care providers should preselect patients for an EIMD on the basis of their experiences.

A strength of this study was the intensive follow-up from students, with more than 900 validated measurements. Although the number of included patients was limited, the number of drug intake comparisons was sufficient to detect omissions in the recorded actuations. However, the small population may have affected the representativeness of the results of the acceptability questionnaire. The statements in this questionnaire were formulated positively or negatively to reduce the risk of positively biased answers. This questionnaire was adapted to this specific EIMD and not validated beforehand. As a consequence, some patients might have experienced difficulties in comprehending the language used or the variety in both positively and negatively formulated questions.

### Implications for the Future

It is recommended that the discussed technical issues should be further elucidated and solved before using EIMD data as an objective adherence measurement, and medication overuse may also be of interest. In order to fully benefit from the EIMD and guarantee reliability and validity, an EIMD should be validated in a setting where the users interact with the system and can encounter technical or user issues. Furthermore, EIMDs and the accompanying self-management program should be evaluated on usability in daily practice. This study provided an example of how to do this. Validation of EIMDs in real-world settings is likely to improve usability in daily practice; the EIMD should be easy to use and measure all actuations correctly, even when the patient is not technically skilled. Future research should pay sufficient attention to different types of patient segments, as the EIMD needs to match with the patient profile (eg, adherence and asthma control), needs, and preferences.

### Conclusions

Comparison of EIMD data with patient-reported inhalations showed that EIMD registrations represented patient inhalations to an acceptable degree, with a PPV of 96%, but these registrations were likely to underreport actual drug intake by 11%. Technical improvements should address the Bluetooth connection and data synchronization. Additionally, patient characteristics contribute to the validity of EIMD measurements, and larger sample sizes are needed to explore their influence. For the acceptance of a self-management program with an EIMD, patients who benefit from self-monitoring and reminders should be targeted by tailoring the possibilities to the needs of the individual user.

## Abbreviations

BMQ: Beliefs About Medicines Questionnaire
EIMD: electronic inhalation monitoring device
PPV: positive predictive value
SUS: System Usability Scale
